# Predicting pediatric diagnostic imaging patient no-show and extended wait-times using LLMs, regression, and tree based models

**DOI:** 10.3389/frai.2025.1652397

**Published:** 2025-09-03

**Authors:** Daniel Rafique, Xuan Liu, Bo Gong, Laura Belsito, Melissa D. McCradden, Mjaye L. Mazwi, Wayne Lee, Graham Ohanlon, Kyle Tsang, Manohar Shroff, Birgit Ertl-Wagner, Farzad Khalvati

**Affiliations:** ^1^Department of Mechanical and Industrial Engineering, University of Toronto, Toronto, ON, Canada; ^2^The Hospital for Sick Children, Research Institute, Toronto, ON, Canada; ^3^Department of Medical Imaging, University of Toronto, Toronto, ON, Canada; ^4^Department of Computer Science, University of Toronto, Toronto, ON, Canada; ^5^Department of Diagnostic and Interventional Radiology, The Hospital for Sick Children, Toronto, ON, Canada; ^6^Australian Institute for Machine Learning, University of Adelaide, Adelaide, SA, Australia; ^7^Women's and Children's Health Network, Adelaide, SA, Australia; ^8^Seattle Children's Heart Center, Seattle, WA, United States; ^9^Institute of Medical Science, University of Toronto, Toronto, ON, Canada; ^10^Vector Institute, Toronto, ON, Canada

**Keywords:** no-show, wait-times, scheduling, prediction, large language model, machine learning, data balancing, calibration

## Abstract

**Introduction:**

Patients missing their appointments (no-shows) are a persistent issue that results in idle resources while delaying critical patient prognosis. Likewise, long waiting times increase frustration for patients, leaving a negative impression on the appointment. In this paper, we explore 3 modalities of diagnostic and interventional radiology appointments for pediatric patients at the Hospital for Sick Children (SickKids), Toronto, ON, Canada. Our goal was to survey machine learning methods that best predict the risk of patient no-shows and long wait-times exceeding 1 hour for scheduling teams to propose targeted downstream accommodations.

**Methods:**

We experimented with 6 predictive model types separately trained on both tasks which included extreme gradient boosting (XGBoost), Random Forest (RF), Support Vector Machine, Logistic Regression, Artificial Neural Network, and a pre-trained large language model (LLM). Utilizing 20 features containing a mixture of patient demographics and appointment related data, we experimented with different data balancing methods including instance hardness threshold (IHT) and class weighting to reduce bias in prediction. We then conducted a comparative study of the improvements made by utilizing continuous contextual data in our LLM which boasted a 51% improvement in F1 score for the wait-time model.

**Results:**

Our XGBoost model had the best combination of AUC and F1 scores (0.96 and 0.62, respectively) for predicting no-show while RF had the best AUC and F1 scores (0.83 and 0.61, respectively) for wait-time prediction. The LLMs also performed well for 90% probability thresholds (high risk patients) while being robustly calibrated on unseen test data.

**Discussion:**

Our results surveyed multiple algorithms and data balancing methods to propose the greatest performing models on our tasks, implemented a unique methodology to use LLMs on heterogeneous data within this domain, and demonstrated the greater importance of contextual appointment data over patient demographic features for a more equitable prediction algorithm. Going forward, the predictive output (calibrated probabilities of events) can be used as stochastic input for risk-based optimized scheduling to provide accommodation for patients less likely to receive quality access to healthcare.

## 1 Introduction

Medical appointment no-shows, often referred to as missed appointments, is a persistent global issue that can prove costly for hospitals (Jabalera Mesa et al., 2017; Dunstan et al., 2023). Additionally, long wait-times due to unoptimized scheduling can negatively impact patient experience and even discourage patients from attending their future appointments. No-shows also have the added risk of increasing complications in patients due to delays in follow-ups. This also results in an overall increased cost in hospital resources to accommodate these events. Although minimizing the costs of no-shows and wait-times are important, it’s critical to be aware of the potential socioeconomic disparity when proposing methods to reduce patient no-shows and long wait-times (Chen, 2023; Taheri-Shirazi et al., 2023).

Approximately 3,000 appointments are scheduled every month for magnetic resonance imaging (MRI), ultrasound (US), and computed tomography scans (CT), within the department of diagnostic and interventional radiology (DIR) at the Hospital for Sick Children (SickKids), Toronto, Ontario, Canada. Due to the large number of DIR appointments conducted every year, even a small rate of no-shows can prove costly. DIR scans play an important role in disease diagnosis, often serving as the initial step in designing treatment plans. Depending on the nature of their health concerns, patients may be directed to various modalities of DIR. Once a clinical department clinician requests a scan for their patient, the request is sent to one of various modalities. If a patient misses their appointment, a letter is then sent back to the referring department to notify them of the occurrence. After the scan is completed, subsequent patient care steps are defined based on the conclusions drawn by the radiologist. This can include communicating whether the patient requires follow-up scans or blood and tissue biopsies before the prescribed treatment plan. Therefore, having accurate predictions for DIR scheduling is critical for minimizing delays in treatment planning and taking steps for equitable delivery of care.

Our research is focused on surveying various predictive models trained on SickKids DIR appointment data to propose the best performing model and methods for clinical use. The future goal would be to utilize this research for a downstream optimization algorithm that uses predicted probabilities for more accurately informed scheduling decisions. Some of these downstream tasks could include providing alternative schedules for patients that have a high risk of missing their appointment, alternative overbooking methods, and proposed targeted accommodations for patients with higher risk of negative appointment outcome. Additionally, we are predicting both tasks (no-shows and wait-times) for the following reasons: the downstream accommodation could utilize both predictions, it serves as a follow up to the results of our previous study (Taheri-Shirazi et al., 2023), our methodologies proposed in this paper are applicable to both tasks using the same data, and the tasks are inherently intertwined problems. This research study provides the first step of maximizing DIR appointment efficiency to ensure an equitable level of care for all patients regardless of patient demographics.

We utilized the following 6 algorithms for each prediction task: logistic regression (LR), support vector machine (SVM), random forest (RF), extreme gradient boosting (XGBoost), artificial neural network (ANN), and a ClinicalBERT large language model (LLM). The major quantitative metrics for success in these experiments are F1 and area under the receiver operating curve (AUC) scores due to the skewed nature of the data. As such, we gave greater weight to performance on the minority class.

Several preventative strategies have been implemented in clinics for missed appointments with varying degrees of success. One category of approach includes intervention strategies which target patient behavior through incentives or deterrents (Vikander et al., 1986), patient prepayments (Garuda et al., 1998), financial penalties for not showing up (Bech, 2005; Goffman et al., 2017), reminder notifications (phone calls, text messages) (Wu et al., 2019; Schwebel and Larimer, 2018), and patient education (Weaver et al., 2019). A systemic review of 29 studies reported that the impact of telephone, SMS, and automated phone calls resulted in a change of 34% in appointments missed (Hasvold and Wootton, 2011). It remains to be seen if these strategies can be further bolstered through targeted reminder notifications where higher risk no-shows benefit from additional reminders. Other strategies include overbooking methods to prevent vacant appointment slots (LaGanga and Lawrence, 2007).

The use of strategies to maximize appointment attendance invokes ethical issues which, if left unaddressed, can result in discrimination, alienation, and stigma. For example, the use of algorithms to predict non-attendance has resulted in overbooking racialized patients, making them wait longer and reducing the quality of their healthcare experience (Samorani et al., 2021). Making a different choice based on the same prediction task, however, could result in improved access to care. Understanding a family’s reasons for potential non-attendance could enable a care team to identify opportunities to address equity-related barriers to attendance, for example: engaging a translator or patient advocate, changing the appointment time to suit a single parent’s work schedule, or providing transportation vouchers.

From our prior research, we found correlations from household income, percent single caregiver, and English proficiency to be significant indicators for no-show status (Taheri-Shirazi et al., 2023). Despite these observations, accurately making predictions on patient behavior and wait-times is yet to be successfully implemented. Our research surveys methods to improve prediction and study the impacts of leveraging appointment specific data over solely using patient demographic features. This paper will first outline our background research and how our data was collected, processed, and analyzed for feature engineering and training. Next, we define the methods and models utilized in our experimentation, including data balancing and calibration. Finally, we will discuss the results of our experiments and the conclusions that can be drawn from them.

## 2 Materials and methods

### 2.1 Literature review

There are numerous factors contributing to patient no-show and wait-time behavior that could point to helpful predictive features in appointment outcome (Daggy et al., 2010; Cronin et al., 2013; Dantas et al., 2018; Joseph et al., 2022). With regards to no-show prediction, factors encompass patient age, distance from hospital, gender, previous appointment history (Lee et al., 2005), socio-economic class, percentage of non English speakers in postal area, referral clinic, income coverages, indication on if the appointment was a follow-up (Lehmann et al., 2007), and appointment specialties. In one study, afternoon appointments with short lead times (time from scheduling to actual appointment date) and long appointment durations were found to influence outcomes of missed appointments (Peng et al., 2014). Additionally, some studies have highlighted the significance of weather conditions on the day of the appointment as an important predictor of no-shows (Peng et al., 2014; Liu et al., 2022).

A systematic literature review published in 2018 revealed that the features most associated with no-show behavior include young adult age ranges, lower socioeconomic status, length of commute, and lack of private insurance. Notably, high lead time and prior no-show history were identified as the features exerting the greatest impact on missed appointments (Mohammadi et al., 2018). Finally, when looking at predictive wait-times, a study on radiation oncology appointments found that the most predictive features were allocated appointment time, radiotherapy fraction number, most recent appointment duration, median appointment durations, and the number of treatment fields (Joseph et al., 2017).

Drawing upon these influential factors, numerous statistical and machine learning models have been proposed to predict patient no-shows and wait-times. Logistic regression has traditionally been the mainstream model for this purpose due to its efficiency in modeling binary responses (Goldman, 1982; Kurasawa et al., 2015; Huang and Hanauer, 2016). However, in recent years, a variety of machine learning algorithms beyond logistic regression have been increasingly utilized, including ANNs and Naive Bayes (Mohammadi et al., 2018). A novel approach introduced in a 2019 study used sparse stacked denoising autoencoders (SSDAEs) for predicting missed appointments with its best model (SDAE + LR) having an AUC of 0.704 and F1 score of 0.288 (Dashtban and Li, 2019). This model integrated data reconstruction and prediction phases, which are typically separated into existing deep learning applications for hospital data which significantly outperformed other methods. This research was followed up again in 2021 with similar results (Dashtban and Li, 2021). Another study done in 2019 compared the performance using recall as opposed to AUC across nine machine learning algorithms including AdaBoost, Logistic Regression, Naive Bayes, SVM, Stochastic Gradient Descent, Decision Tree, Extra Trees Classifier, Random Forest, and XGBoost (Joseph, 2019). It was found that AdaBoost outperformed all other algorithms with an AUC of 0.7. In contrast, a systematic literature review of machine learning techniques applied to no-show appointments done in 2022 across 24 articles showed that RF had the best performance with an AUC of 0.969 (Salazar et al., 2022). We see a similar trend with regards to wait-time prediction models in which RF models performed the best, as shown in a radiation oncology study done in 2017 (Joseph et al., 2017). Another study by the Oregon Health Science University (OHSU) conducted research on outpatient pediatric ophthalmology appointments to predict wait-times using a variety of ML algorithms in which RF achieved the highest root mean squared error (RMSE) and the highest AUC score of 0.811 (Lin et al., 2020). Similar patient demographic features were used to achieve these performance metrics such as age, financial class, returning patient, and more.

Finally, since our data faces the added challenge of class imbalance, it was important for our research to consider methods that mitigate any bias in its output. A pediatric hospital in Chile tackled the class imbalance problem through algorithms such as RUS Boost, Balanced RF, Balanced Bagging and Easy Ensemble. They concluded that the imbalanced learning ensemble methods outperformed the basic scikit-learn algorithms when predicting across most departments (Dunstan et al., 2023). Another study done in 2024 implemented a Symbolic Regression (SR) algorithm to predict no-shows and addressed class imbalance by applying a resampling technique: Instance Hardness Threshold (IHT) (Deina et al., 2024). Their results indicated that SR with IHT had superior performance over more traditional techniques on this problem.

### 2.2 Data

This section covers the data received from the SickKids DIR department that was collected using EPIC databases for our 2 prediction tasks. The dataset characteristics, exploratory data analysis, visualizations, cleaning methodology, feature engineering, and text augmentation for building LLMs are outlined below for replication of our model implementations.

#### 2.2.1 Dataset

The raw data collected from DIR included 421,743 rows and 67 features. The original dataset contained samples from June 2018 up to and including December 2023. This dataset contained 130,975 unique patients with 3 major categories of appointments which are completed, canceled, and sent statuses. We only used completed outpatient appointments due to inconsistencies in the other categories that would require extensive auditing. Additionally, the raw data contained 134,038 MRI, 244,284 US, and 44,107 CT diagnostic scans which had varying rates of long wait time and no shows. The patients used for our study range from ages 1 day old to 6,574 (18 years) days old and all live within the postal regions of Ontario beginning with the following letters: L, M, N, P, and K. All models were tested on the same unseen test set containing 16,277 instances from June 2023 to December 2023 (the last 6 months of the dataset) to prevent biases and data leakage.

#### 2.2.2 Exploratory data analysis

This section defines some key data characteristics that better showcase the patient demographics these models were trained on.

Firstly, the data was relatively evenly split among the two sexes with males representing approximately 54% of the data and 53% of all missed appointments. Next, wait-time was found to have a mean of 31.65 minutes. This metric was derived from the difference between the exam start time and patient check-in time. After dividing the distribution of wait-times into bins of 60 minutes, we found that most of the wait-times fall within 1 hour, which allowed us to create an approximate threshold for defining a long wait-time and excluding outliers which we will explore further in section 2.2.3.

The procedure category is another fundamental aspect of each appointment with it being either an MRI, US, or CT scan. Our analysis found that approximately 28% of appointments were for MRI scans, 63.6% for US, and 8.4% for CT. Expectedly, the bulk of the no shows came from US appointments which made up 90% of the no-show instances, followed by 6% for MRI and 4% for CT. Additionally, US had the highest rate at 2.1% no-show, followed by CT at 0.66%, and MRI at 0.30%.

**Figure 1:**
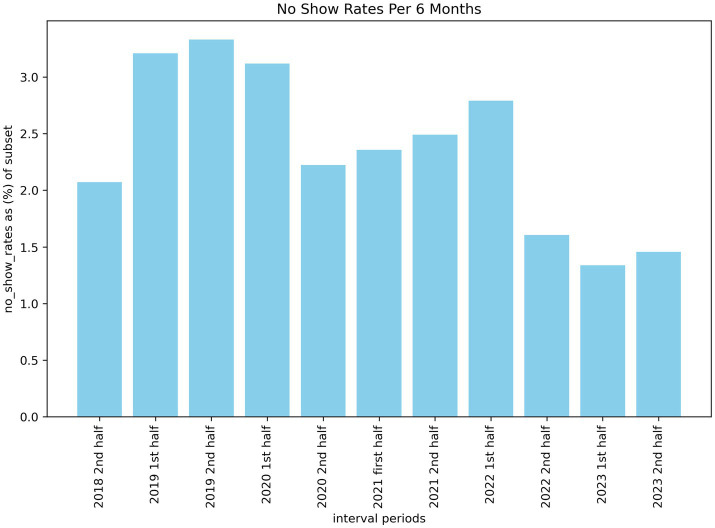
Bar plot visualizing the rate of no shows biannually.

When investigating the number of missed appointments across 6-month intervals, we can see a higher number of no-shows a few years ago compared to the previous 18 months. Figure 1 illustrates this trend from 2018 to 2023 with a statistically significant drop (*p* < 0.05) from the first half of 2021 to the second half of 2022. The reason for this drop is not formally known, however, our estimate is that it may be correlated with the removal of covid restrictions within Ontario around the time of the drop. This may have incentivized patients to follow through on their appointments as it was also around the time that post-pandemic wait-lists began to increase. This meant that missing an appointment could have pushed out re-booked slots by weeks or even months.

Another important indicator for predicting both no-shows and long wait times is the appointment schedule hour (Peng et al., 2014). We categorized the schedule hours into 4-time frames which are early morning (12 am - 6 am), morning (6 am – 12 pm), afternoon (12 pm – 5 pm), and evening (5 pm – 12 am). When analyzing the pre-processed dataset of 154,935 appointments, we found that approximately 55% of scheduled exams were in the morning, 35% in the afternoon, 10% in the evening, and <0.19% in the early morning. Of the appointments resulting in no-show, 0 were from early morning, 352 were morning appointments (0.41% no-show rate), 3,083 were afternoon appointments (5.69% no-show rate), and 158 (1% no-show rate) were evening appointments. Therefore, based on our analysis, afternoon appointments are proportionally the highest risk of resulting in a missed appointment. It is important to note that the appointment dates for no-shows are the time that staff indicated within the system that the patient missed their appointment (overwriting previous dates) and not the exact appointment time. For this study, we assume that the time indicated for patient no-show is approximately the same as the original appointment date.

Based on our literature review, lead times were one of the most important features for no-show prediction models (Peng et al., 2014). Unfortunately, within our data, lead times are only available for completed and attended appointments due to the EPIC system workflow. Currently, if a patient misses their appointment, a duplicate of the original order is made in the system with no-show related tagging and IDs that release the order as the same day as the final schedule date. This makes every exam date the same as the date the appointment was entered into the system as a no-show (i.e., lead time equals 0). Retrieving the original order information would reveal the actual lead time, however, this required an extensive audit and more resources than we had available for this study.

Finally, we ran a correlation test using a heatmap across prominent numerical features to find relationships that may exist between our potential feature set. Our correlation heatmap revealed a slight correlation between schedule hour, previous no show history, and the no-show label. Additionally, scheduled hour, wait-time-information-system priority (WTIS-Priority), and wait-time features appeared to have higher correlations.

#### 2.2.3 Data cleaning

This section outlines 9 steps we took to pre-process our data and their associated justifications. We also removed any fields that may provide unfair knowledge of appointment data that would not be available at the time of scheduling. The raw data included 421,743 ordered appointments with 67 features and 130,975 unique patients. All patients in our dataset were anonymized with unique identifiers for patient data privacy.

*Step 1—filter for only complete appointments—remaining data = 271,802.*

The first step in our data cleaning process was dropping any incomplete appointments using the “procedure status” field. After discussions with the DIR department, it was best to only keep “complete orders” since other order categories were inconsistent in their labeling of no-show such as with canceled orders. The resulting appointment count then became 271,802.

*Step 2—filter out appointments that had a missing check-in datetime—remaining data = 271,772.*

We dropped any orders that had a missing check-in datetime because all complete appointments, including those that were missed, should have had a logged check-in time according to the DIR department. Even when a patient misses an appointment, the system replaces the check-in date with the missed exam date. This resulted in a total appointment count of 271,772.

*Step 3—filter out non-Ontario postal codes—remaining data = 267,423.*

We filtered our data to only include postal codes beginning with K, L, M, N, and P such that our models target a certain range of Ontario that would also have census statistics. This resulted in 267,423 remaining appointments.

*Step 4—filter out patients over the age of 18—remaining data = 262,267.*

We filtered out any patient age at the time of appointment that was over 18 (6574.32 days) to focus our research on pediatric DIR exams which created an appointment count of 262,267.

*Step 5—filter out patients without a listed sex—remaining data = 262,162.*

We removed any patients that did not have a specified sex (male or female) for uniformity of training data, resulting in 262,162 patients.

*Step 6—Filter out any patients who had a wait-time over 300 min—remaining data = 253,497.*

We removed any scans that had a wait-time (check in time to exam start time) lasting over 300 minutes since any appointment over this amount was an egregious outlier that was assumed to be an anomaly not properly modelled in our data. This resulted in a total of 253,497 orders.

*Step 7—Filter out appointments with durations longer than 5 h—remaining data = 252,718.*

Appointments were filtered to only include appointment durations (exam start time to exam end time) between 0 and 5 hours as we assumed these were outliers not properly modelled in the data considering clinics only operate for 8 hours. This left a total of 252,718 remaining appointments.

*Step 8—Filter out duplicate orders based on matching exam datetimes and accession numbers—remaining data = 157,200.*

When analyzing the data we discovered that there existed instances of duplicate orders. These included appointments where a patient had the same check-in, ordering, exam, and schedule date time. Alternatively, there may be appointments with the same accession number. These instances meant that a patient had multiple appointments scheduled for the same time slot. Some practices also split an exam resulting in what looked like multiple appointments. We removed these duplicates as well since a no-show would occur on all split order entries. Removing duplicates resulted in 157,200 remaining orders.

*Step 9—Filter out orders that did not have a referring clinical specialty—remaining data = 154,935.*

Finally, we mapped web-scraped clinical specialties to the ordering provider and removed any appointments that did not include a clinical provider specialty resulting in a final total of 154,935 orders.

After cleaning, additional columns were added as numerical encoded versions of categorical variables such as schedule month (1–12), scheduled day of week (1–7), and gender (0, 1) (see Appendix for final feature list). The data was then split into training and testing such that the unseen test set would include all appointments from the last 6 months of our data (June 1, 2023, to December 2023 inclusive). Our final training set for baseline models (models trained on data without any under sampling techniques) comprised of 138,658 appointments and a test set of 16,277. During model training, all models except for the BERT LLMs (which used a 20% validation split of the training set), utilized 10-fold cross validation with a split ratio of 9:1 on training data.

#### 2.2.4 Feature engineering

Several of the features have been engineered from existing features and external sources. One set of features included estimated patient demographic information by postal code which was gathered from Statistics Canada 2021 census. This means that the few patients that reside outside the Canadian census were not included in our predictive modeling as discussed in section 2.2.3. Demographic results were gathered based on postal codes starting with L, M, N, P, K. The average statistics for postal areas included were the percentage of English speakers, percentage of single-parent households, and average income. Next, missing distance information was imputed using the residing postal code and a calculated distance to the hospital based on the haversine formula. Another significant feature engineered for prediction was the estimated referring department. Using the authorizing provider of the patient appointment, we scraped the clinical specializations of each doctor and nurse practitioner from the college of physicians and surgeons of Ontario (CPSO) (CPSO, 2019). These were then assigned as the patient’s authorizing category. After scraping over 3,750 doctors, we narrowed down the specializations to 41 categories with the aid of a clinician. So, if a patient’s authorizing provider had a specialization in cardiology, the estimated referral specialty would be cardiology.

For our BERT LLM models, we took all the relevant fields from the tabular data and transformed them into a single interpretable string resembling text input. A script to take key numerical values and translate it into a meaningful sentence was created to leverage transfer learning and the attention based contextual learning of LLMs (see Appendix for a sample input for an LLM model). An additional sentence was also added on select LLM experiments that included the reason for exam. This feature was not available for a large portion of the target class of data for no-show prediction and was thus omitted in the base models. Additionally, any reason for exam that had no-show tagging within it was scrubbed, resulting in a much smaller usable subset for that feature. Table 1 summarizes our feature engineering.

**Table 1:** Engineered features and the source information used to generate them.

Engineered features	Available information
‘Age at scan’	Patient’s age at scan check in date and time, Date of birth
Postal code’: first three digits of FSA	Postal code
‘Procedure name’: procedure names (MRI, US, CT)	Procedure category
‘Week day’: appointment week day ranging from 1 to 7 representing Monday to Sunday	Scheduled exam date and time
‘Scheduled hour’: scheduled hours ranging from 1 to 23	Scheduled exam date and time (positive no shows are date exam entered as no-show and assumed to be scheduled exam date)
No show’: appointment no show (no show: 1, show:0)	Procedure name
‘Distance’: distance to hospital	Postal code
‘Pre appointments’: accumulative summation of the patients’ previous appointments	Patient MRN
‘Pre no show’: previous no show history	Patient MRN, no show
‘Income’: the average household income based on postal	2021 Canada Census of Population
‘Non eng %’: percentage of non-English speakers in patient’s neighborhood	2021 Canada Census of Population
‘Single parent %’: percentage of single parent families in patient’s neighborhood	2021 Canada Census of Population
Authorizing Category: the estimated ordering providers specialty	Authorizing Provider and CPSO public data on doctor specialties
Impute missing WTIS-Priority entries: wait time information system priority which we assume to be the priority level assigned. (US all given value of 0 since these do no have a priority)	Missing WTIS-Priority levels were imputed using a k-nearest neighbors method using the 20 nearest neighbors.
‘Appointments in hour’: the number adjacent appointments that occur that the time of a specific appointment	Scheduled exam date and time was used to group all exams that started within the same hour and quantify the adjacent appointments
Numerically encoded features	All features with categorical strings were converted into sequential numerical encoding. For instance, gender having male and female was converted to 0 and 1.

### 2.3 Models

The following section describes the 6 underlying architectures we used for prediction and the steps taken to train, validate, and test them. All models were tested using the last 6 months of data to assess the model’s generalizability on time series data. Additionally, models were trained and tuned using a random seed of 42 for replication.

#### 2.3.1 Logistic regression

Logistic regression is a binary classification technique that maps a weighted set of features using a logistic function such as a sigmoid, to a set of probabilities that can be interpreted as classifications. The model was tuned using 10-fold stratified cross validation with an assigned class weighting of 10:1 for the positive minority class to account for data imbalance. The solver was selected to be Newton Cholesky after conducting a grid search for optimal solvers which creates a hessian matrix and solves the linear system. Finally, the maximum iterations before converging were set to 1,000 for computational efficiency. All data entered had to be scaled and encoded into numerical format for training and validation.

#### 2.3.2 XGBoost

Gradient boosting is an efficient machine learning algorithm that ensembles additive weak learner models to sequentially correct the previous decision trees by following the gradient to minimize loss (Chen and Guestrin, 2016). Our XGBoost model uses a log loss metric to calculate error and uses a weight of 10:1 for the minority class to accommodate for the class imbalance selected through grid search. Finally, the model was validated using 10-fold stratified cross validation and fitted on scaled numerical representations of our feature set.

#### 2.3.3 Random Forest

A random forest model utilizes a decision tree system that ensembles and aggregates multiple small decision tree models to make a prediction. The RF was tuned using 10-fold stratified cross validation which had an optimal weighting of 5:1 for the minority class. All other parameters were left unchanged based on our validation grid search.

#### 2.3.4 Support vector machine

SVM is a classic prediction model that uses support vectors to maximize the margin between the ‘*w’* hyperplane that separates classes and its support vector to improve classifications. SVMs typically do not perform well on large datasets due to slower computational time. However, they were used as a baseline model for their ability to identify small patterns in complex datasets. The SVM used stratified cross validation with a split of 10 folds on numerically encoded normalized data. The model was built using the SVC library with a class weight of 10:1 for the minority class as this was found to be optimal by our grid search.

#### 2.3.5 Artificial neural network

A feed forward ANN was used to discover if deep learning could be beneficial to our prediction problem as hypothesized in our previous paper (Taheri-Shirazi et al., 2023). While deep learning has made significant advancements in prediction and generative modelling, it is well known that they typically struggle with non-homogenous tabular data such as ours (Shwartz-Ziv and Armon, 2022). Despite this, we wanted to explore the potential benefits these models could have by overcoming the limitations of our feature engineering. Our NN is comprised of 2 dense layers (64 and 32 units respectively) with RELU activation functions. The output dense layer produces 1 binary classification and uses a sigmoid activation function. The model uses an Adam optimizer with a learning rate of 0.001. The loss function for our model is a custom loss function that weighs false positives and negatives in accordance with the imbalanced data. False positives are given a weight of 2 and false negatives a weight of 3. The function then uses binary cross entropy loss weighted as 1 and then computes the number of false positives and false negatives in a batch. The final loss function is the weighted sum of the binary cross entropy loss, false positives, and false negatives. The final model was validated with 10-fold stratified cross validation over 10 epochs with a batch size of 32.

#### 2.3.6 Bert LLM

To leverage the advancements of transfer-learning, we implemented an LLM that would capture the context of sequential text data. BERT models use bi-directional encoding to read an entire sequence at once and obtain a word’s surrounding context. A linear classification layer is then added on top of the transformer for binary prediction. Having created an ML pipeline that transforms the tabular data into an interpretable text string that selectively places greater attention on certain features, our BERT model then transfers its initial weights from a clinically specialized version of BERT called “Bio_ClinicalBERT” from Hugging Face. The LLM was trained on 5 epochs using a 16 gigabyte Nvidia Tesla P100 HPC GPU with an AdamW optimizer, a learning rate of 3e-5, and a warmup using the first 10% of the training data. This was validated using a separate validation set that is 20% of the original train data for fine tuning before being tested.

### 2.4 Imbalanced learning techniques

Imbalanced Learning attempts to resolve severe class distribution skews. Without applying imbalanced techniques, an algorithm might become biased towards the majority class resulting in poorer performance on the target event (no-show and long wait-time) (Hasanin et al., 2019).

Many model pipelines have been proposed to handle class imbalance which are commonly used such as data level techniques where the data is either under sampled (Beckmann et al., 2015; Smith et al., 2013) or over sampled (Chawla et al., 2002) to balance the classes. Although data level techniques are widely used, they often suffer from the issue of generalizability where the training data does not reflect real world proportions. Other methods include algorithm level ensemble techniques. An algorithm level technique forces the algorithm to pay more attention to the minority class. Ensemble level techniques make conventional ensemble algorithms more sensitive to the minority class such as RUSBoost (Seiffert et al., 2010) and balanced RF (Chen et al., 2004).

For the purposes of our research, we experimented with a data level technique called Instance Hardness Threshold. It generates Instance Hardness to filter instances that are likely to be misclassified by multiple classifiers. An instance’s Hardness is evaluated based on how many classifiers misclassify it. Once a hardness threshold was established, instances with hardness values exceeding this threshold were excluded. Since the LLM models used an augmented continuous version of the same numerical tabular data, the weak learner models found many of the instances as hard. This resulted in an overly aggressive under sampling of the majority class. So, we decided to omit this method from our final evaluation of the LLMs. Additionally, we tuned all our default models to pay more attention to the minority class and type II errors through grid search class weighting to account for class imbalance without manipulating the data in training.

#### 2.4.1 Model calibration

A clinically deployable model should output a continuous risk metric that would allow scheduling teams to implement different contingencies for the patient appointment rather than having a simple binary classification (Jiang et al., 2011). A common approach is to utilize the predicted probabilities associated with each classification as a risk measure. However, these probabilities can vary greatly depending on the algorithm and do not accurately reflect the true state of the prediction. For instance, XGBoost tends to be overconfident in its predictions. So, we used isotonic regression calibration on the validation data to ensure the prediction probabilities have a linear relationship with true event rates. Additionally, isotonic regression is seen to improve performance on imbalanced datasets which typically outperform other parametric calibration methods (Huang et al., 2020).

To calibrate the models with default data balancing, we saved the predicted probabilities and labels during each split of cross validation. During each training fold, the model was trained on 90% of the data while predicting for the other 10%. So, when combining the predictions of the 10% splits, we produced a set of validation probabilities equivalent to the training set size. For the LLM models, the regular 20% validation split predictions were used for calibration. Using these prediction probabilities, we then calibrated the validation probability to fit a perfectly calibrated line (positive diagonal line) using isotonic regression. The fitted calibrator was then used for the final test predictions to provide generalizable calibrated probabilities.

## 3 Results

In this section, we discuss our experimental results applying the models presented in section 2. We first compare our calibrated results without any data balancing techniques besides class weighting followed by the IHT method to under sample 5 of our models for both no-show and long wait time classification tasks. We then present results comparing our LLM’s utilizing a subset of the data that includes a more extensive contextual feature called “reason for exam.” Finally, we compare the results of our models using a 90% probability threshold for well calibrated models to assess the precision of predictions with a high risk of a target event.

### 3.1 No-show model results

**Figure 2:**
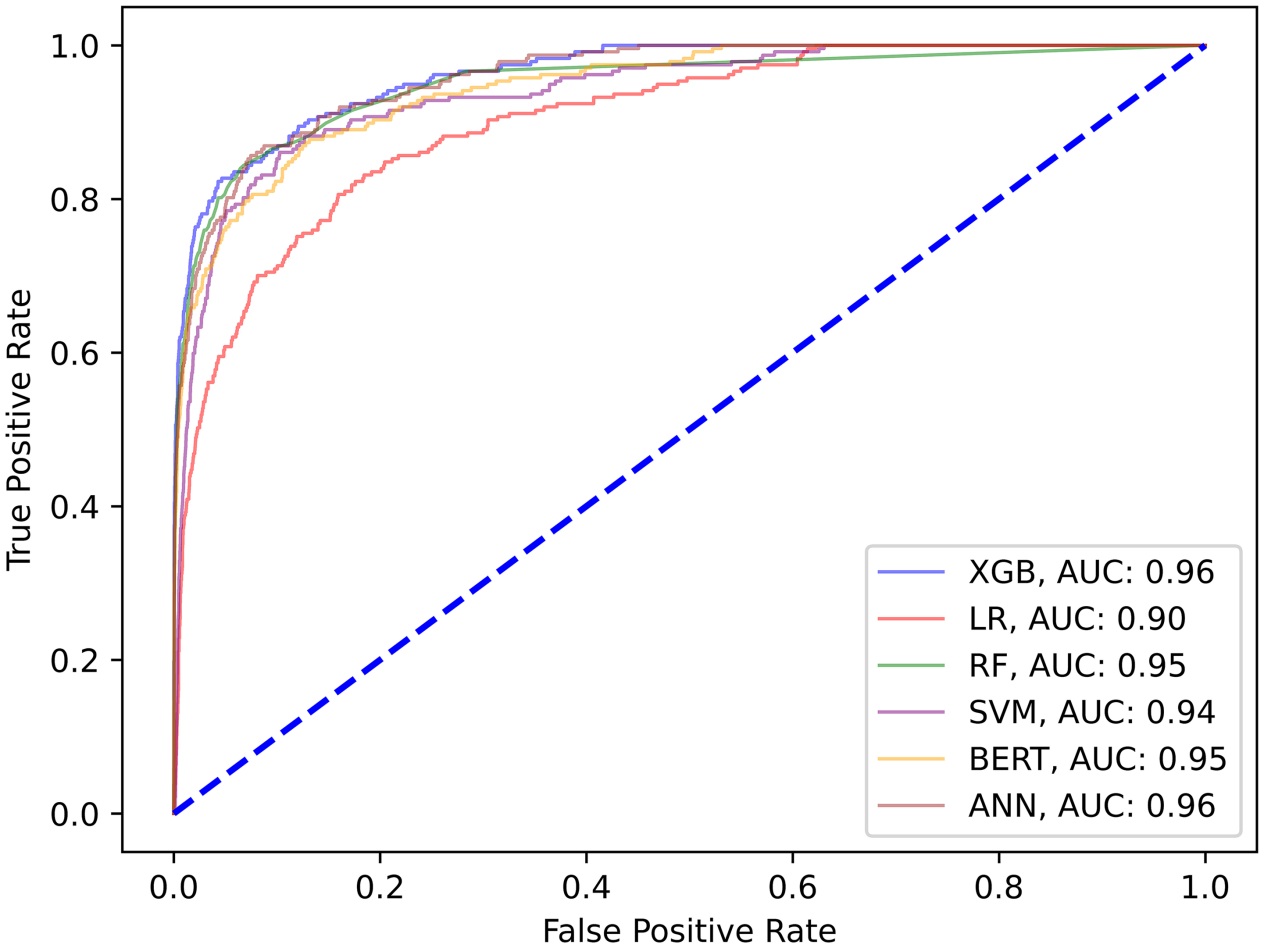
ROC curve on test data for all calibrated default balanced no-show models.

Our first set of models were trained to predict patient no-shows using a mix of demographic and appointment related features. Our initial results shown in Table 2 provide the relevant performance metrics across our 6 chosen models using the default balancing for no-show across all modalities. Our baseline approach is to adjust the class weights and tune the parameters using grid search to penalize the minority misclassifications more heavily. Figure 2 shows the receiver operating curve for all default balancing no-show models on testing data. The second approach uses the IHT method on top of the baseline approaches with results shown in Table 3. Finally, the test data for the LLMs take the same input as the other models but transforms them into continuous text strings.

**Table 2:** No-show results—all modalities with default data balancing.

Model	AUC	F1	Precision	Recall	Accuracy
XGBoost	0.96	0.62	0.82	0.49	0.99
Logistic regression	0.90	0.34	0.38	0.32	0.98
Random forrest	0.95	0.60	0.68	0.54	0.99
SVM	0.94	0.33	0.45	0.26	0.98
BERT LLM	0.95	0.51	0.79	0.38	0.99
ANN	0.95	0.57	0.80	0.44	0.99

**Table 3:** No show results—all modalities with IHT under sampling.

Model	AUC	F1	Precision	Recall	Accuracy
XGBoost	0.87	0.34	0.22	0.78	0.96
Logistic regression	0.91	0.39	0.19	0.64	0.96
Random forrest	0.94	0.37	0.25	0.72	0.96
SVM	0.83	0.21	0.12	0.73	0.92
ANN	0.92	0.14	0.08	0.82	0.85

### 3.2 Wait-time model results

**Figure 3:**
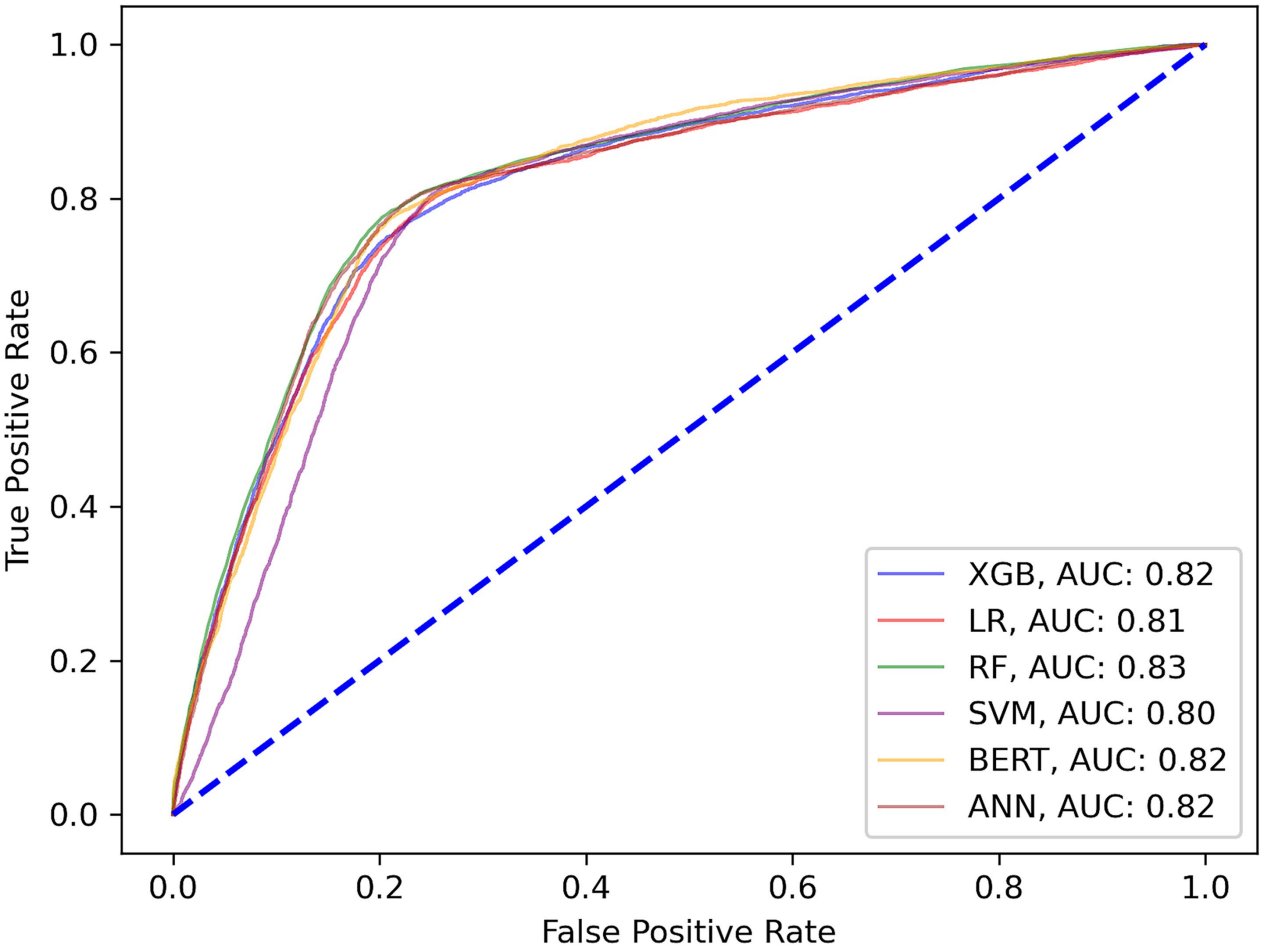
ROC curve on test data for all calibrated default balanced wait-time models.

The following set of model results is for our binary wait-time predictions where class 1 is for appointment wait-times exceeding 1 hour and class 0 is for wait-times less than 1 hour. Figure 3 plots the ROC curves for each model on the wait-time task. Next, the results provide an additional layer of insight with regards to the performance methodology of using IHT on skewed data. Table 4 reports the calibrated balanced data results across our models while Table 5 reports our results using the IHT method. As with the No-Show models, the BERT models did not utilize an IHT method of class rebalance. However, the default BERT model reported here used the reason for exam feature as it produced superior results (discussed further in Section 3.3).

**Table 4:** Wait time results—all modalities with default data balancing.

Model	AUC	F1	Precision	Recall	Accuracy
XGBoost	0.82	0.57	0.61	0.54	0.80
Logistic regression	0.81	0.57	0.61	0.54	0.80
Random forest	0.83	0.61	0.62	0.60	0.81
SVM	0.80	0.53	0.55	0.52	0.77
BERT LLM	0.82	0.62	0.59	0.66	0.79
ANN	0.82	0.62	0.60	0.65	0.80

**Table 5:** Wait time results—all modalities with IHT under sampling.

Model	AUC	F1	Precision	Recall	Accuracy
XGBoost	0.82	0.49	0.33	0.95	0.5
Logistic regression	0.81	0.56	0.41	0.88	0.65
Random forest	0.81	0.55	0.40	0.89	0.63
SVM	0.77	0.62	0.5	0.82	0.75
ANN	0.82	0.62	0.5	0.82	0.75

### 3.3 Appointment context feature LLM results

The “reason for exam” field is an additional text feature available within our data that provides clinical comments on why the patient was scheduled for an exam. Since these are unstructured text strings, the utilization of this feature was best reserved for an LLM. As mentioned in section 2.0 of our methodologies. When curating this feature, since it was only available on a fraction of the positive classes for no-shows, we decided to create an additional experiment on a smaller subset of train and test data reflective of the raw data proportions. Additionally, since missingness was not a limitation for the wait-time models with both classes having reason for exam fields, we provide comparative results using the same data with and without the field. The goal of this experiment is to show the importance of appointment specific context when predicting patient behavior. The following Tables 6, 7 show the performance of our LLMs with and without the “reason for exam” feature for predicting no-shows and long wait-times, respectively. Additionally, these tests were conducted with tuned class weights and default data balances. It is important to note that when comparing the results of the no-show models, the LLM with the reason for exam feature has a significantly smaller dataset with 32 positive cases on a test set of 2,207 and a total train size of 15,257.

**Table 6:** No-show LLM results—all modalities with and without “reason for exam” field.

Model	AUC	F1	Precision	Recall	Accuracy
BERT LLM without reason field (full dataset)	0.95	0.51	0.79	0.38	0.99
BERT LLM with reason field (smaller subset)	0.86	0.10	0.25	0.06	0.98

**Table 7:** Wait-time LLM results—all modalities with and without “reason for exam” field. (full training dataset used for both models with 20% validation split).

Model	AUC	F1	Precision	Recall	Accuracy
BERT LLM without reason field	0.82	0.41	0.66	0.30	0.79
BERT LLM with reason field	0.82	0.62	0.59	0.66	0.79

### 3.4 Results for predictions with high probability of target class

This section assesses performance when taking the prediction probability as a risk measure. A simple binary prediction regarding a no-show would not be as practical in clinical use because different response protocols would have varying levels of impact (Jiang et al., 2011). For instance, if a model gives a high probability of no-show, then a scheduling team may want to utilize more invasive protocols such as overbooking. However, this would mean false positives are more costly which would require a model with high precision. In contrast, if the model has a smaller probability of patient no-show, then less extreme protocols such as additional personal reminder calls could be suggested. The following results in Tables 8, 9 presents model performances when looking at patients with over 90% probability of no show and long wait-time. Additionally, each model was predicted on the same test set of 16,277 with a total of 237 positive instances. The precision evaluation metrics are based on predictions with greater than 90% probability of the target class and not overall predictive accuracy using the threshold. This is the number of true positives predicted with above 90% probability divided by the total number of predictions above 90% probability.


$$\text{precision}=\frac{\mathrm{TP}}{\mathrm{TP}+\mathrm{FP}}$$

**Table 8:** Calibrated No-show model precision with default data balancing on test set for predictions with probability of no-show greater than 90% (all predictions are positive).

Model	Total number of high probability predictions	Number of true positive predictions	Precision
XGBoost	96	89	0.93
Logistic regression	4	1	0.25
Random forest	96	82	0.85
SVM	0	NA	NA
ANN	75	68	0.91
BERT LLM	64	59	0.92

**Table 9:** Calibrated wait time model precision with default data balancing on test set for predictions with probability of wait-time greater than 90% (all predictions are positive).

Model	Total number of high probability predictions	Number of true positive predictions	Precision
XGBoost	102	88	0.86
Logistic regression	4	3	0.75
Random forest	53	51	0.96
SVM	0	NA	NA
ANN	7	4	0.57
BERT LLM	132	127	0.96

**Figure 4:**
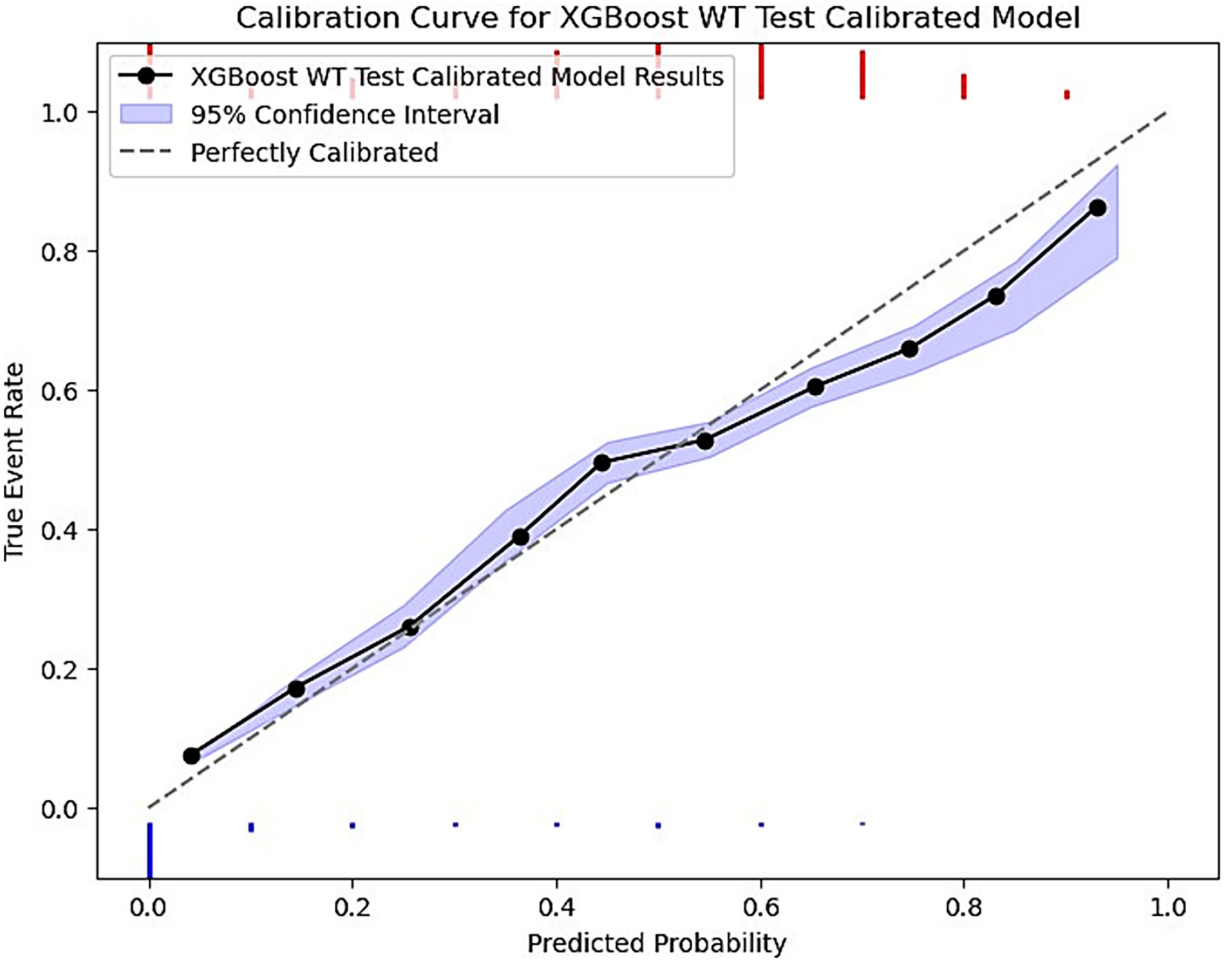
XGBoost calibrated model prediction plot of predicted probability vs. the true event rate. The plot groups data into 10 bins. The purple ribbon indicates the 95% confidence interval of predictions using bootstrapped resampling. The blue histogram at the bottom represents the density of negative events (majority class) and the red histogram at the top is for the positive events (minority class).

Finally, each model was calibrated using isotonic regression on the validation data, however, some models were still not well calibrated to the unseen test data. These included the LR, SVM, RF no-show models, and the ANN wait-time model. The remaining models were relatively well calibrated to the test data, thus allowing us to use the calibrated probabilities as a risk metric. Figure 4 is a sample calibration plot of the XGBoost wait-time model.

### 3.5 Feature importances

**Figure 5:**
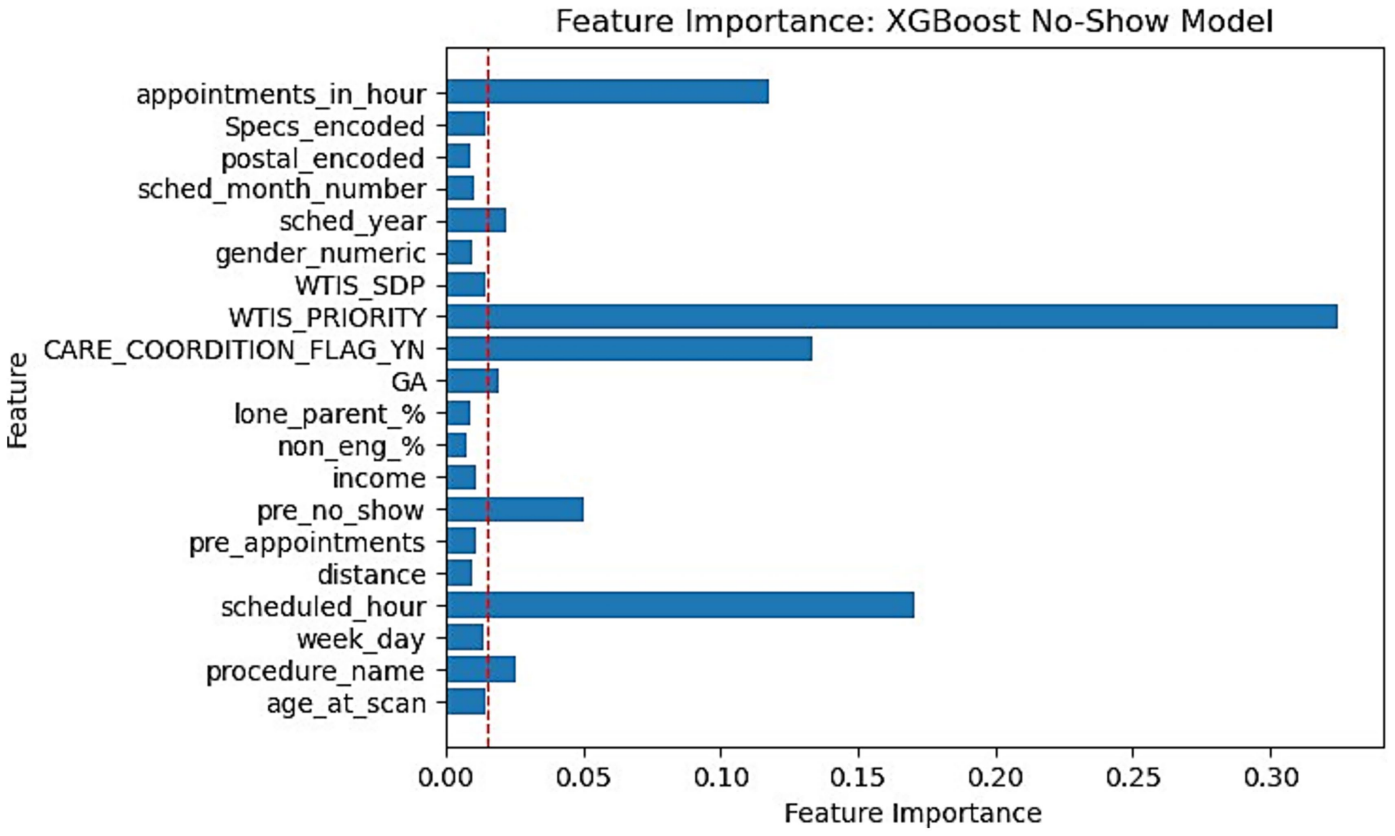
Feature importance bar chart for XGBoost on No-Show prediction. Red dotted line marked at 0.015 weight to differentiate relative lower importance features.

**Figure 6:**
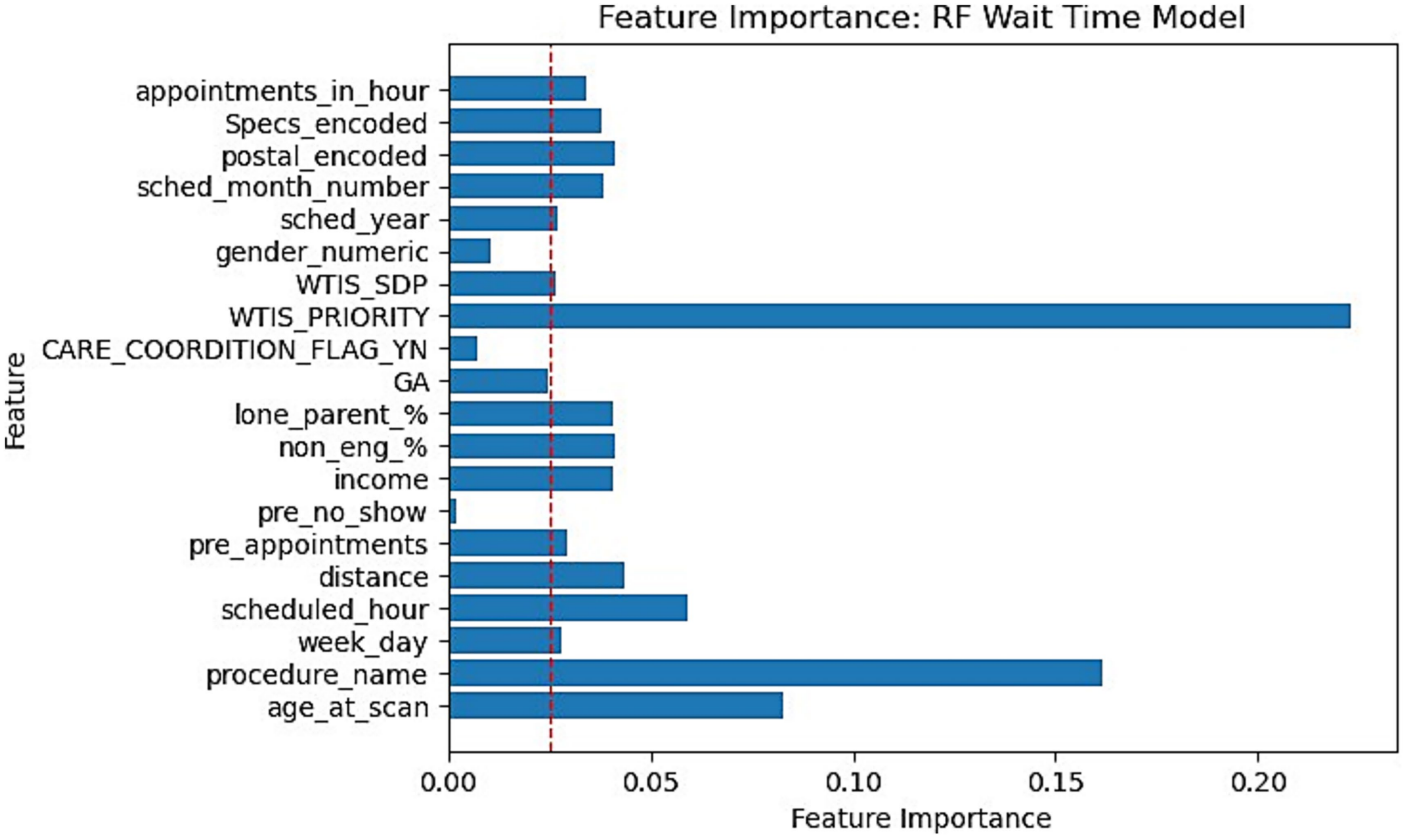
Feature importance bar chart for RF on Wait-time prediction. Red dotted line marked at 0.025 weight to differentiate relative lower importance features.

This section reports the feature importance metrics of the XGBoost and RF models which were the most well-rounded for no-show and wait-time tasks, respectively. The following Figures 5, 6 plot the feature importances on a scale of 0 to 1 in which higher weights are given greater importance within predictions.

## 4 Discussion

Within this research study, we experimented with a broad range of machine learning architectures for 2 prediction problems which are patient no-show and long wait-times. This research follows up on our previous study (Taheri-Shirazi et al., 2023) by providing more insight into new methodologies of predicting these appointment outcomes while improving upon our previous results. The novelty of our research comes from the uniquely large dataset specialized for pediatric DIR appointments, proposed methodology for leveraging deep learning on these tasks, comparatively studying model performances with contextual feature sets, and developing a set of robust risk-based models that boast promising performance for high probability thresholds.

Firstly, as our experiments were able to leverage more data, virtually every model outperformed our previous study when comparing models trained on all modalities. Previously, although the AUC score had a promising value of 0.8, the F1 scores were lacking with a score as low as 0.16 for the no-show model and 0.43 for wait-time prediction (Taheri-Shirazi et al., 2023). In contrast, our best no-show model, the XGBoost, achieved an AUC and F1 score of 0.96 and 0.62, respectively. We also found that for no-show models, performance on US appointments had improved F1 scores but similar AUC scores. This is likely due to the large skew of appointments being US, with the majority of no show cases being from this modality. Alternatively, wait-time models seemed to perform much better on CT and MRI exams, which is likely a result of a much larger skew of positive classes being from MRI and CT scans.

Next, the literature typically used tree-based models for this problem due to its structured data, however, our deep learning models still performed comparatively well given the nature of the data. This would prove very promising since the feature set could be expanded from its current state to better leverage the deep learning architectures. This was especially apparent with the LLM wait-time models. The BERT wait-time model had an almost equivalent performance to the best performing RF model while also being well calibrated and having the best precision when using a high probability threshold which is more relevant for the downstream use case of these algorithms. Although we expected to see poorer performance due to the nature of our heterogenous data, our LLM models achieved an AUC score of 0.95 for no-show and 0.82 for the wait-time model. While we tuned the model and prompts to yield a good result in validation, there is still significant room for improvement with regards to using LLMs for this use case given additional contextual data.

The “reason for exam” and “clinician specialties” are among 2 fields that we expected to provide a unique context to improve classifications. The results of our comparison in LLM performance with and without the “reason for exam” feature indicated that this contextual feature is significant as it was able to improve our F1, recall, and accuracy score by a substantial amount for wait-time tasks (51%, 122%, 0.38% respective improvement). However, since our no-show data with a valid “reason for exam” was limited to only 354 total positive cases in training and 32 in testing, the results were expectedly low for F1, recall, and precision. Despite this, the AUC score of our smaller no-show model still achieved a value of 0.823. These results demonstrate not only how we can leverage the advantages of LLMs for these tasks but also the significant gain in performance from contextual data. This allows our models to be less dependent on patient demographics, which is critical for equitable decision making when implementing accommodation or alternatives for high-risk individuals. This is because the predicted risk would instead be based on a patient’s appointment and scheduling circumstances rather than personal demographics such as income.

Next, contrary to the literature, our models did not perform better when utilizing the IHT method. The performance metrics for most models were either relatively the same or significantly worse. This may have been a consequence of overly aggressive under-sampling by the algorithm on the majority class. The weak learners remove too many samples that are close to the decision boundary and deem it as hard to classify. This may have resulted in reduced generalizability and the model’s ability to learn distinctions between classes. Additionally, IHT typically uses linear classifiers as weak learners while our data likely requires more complex non-linear decision boundaries. A better approach might be to introduce an under-sampling threshold and utilize more complex weak learner models to classify hard cases to limit the removal of too much data. Additionally, combining this with minority over sampling techniques such as SMOTE could improve robustness as studies have shown this can improve generalization (Kim and Jung, 2023).

Feature importances were another significant finding within our research. Our XGBoost and RF feature importance indicated that WTIS-Priority was one of the most significant features for both no-show and wait-time prediction as shown in Figures 5, 6. However, the WTIS-Priority is possibly a redundant feature for US appointments (majority) as they are all valued at 0 priority since this was not a field used by the modality. US appointments having superior no-show prediction performance in contrast to CT and MRI cases may point to a possible grouped modality (US vs. MRI/CT) feature being a significant indicator in prediction over the priority level for no-shows. Additionally, “Care coordination flag,” “scheduled hour,” “scheduled in hour,” and “previous no-show” are other sources of higher feature importance for the no-show XGBoost model and the wait-time RF model. Among the patient demographic features, coinciding with the literature, we found that socio-economic status portrayed through income is the most prominent feature, but only slightly more than the other features. However, these features prove to be less significant in comparison to appointment specific fields such as general anesthesia, clinician specialty, and procedure name (modality). This could suggest that future models may still perform well while omitting these controversial features if more relevant appointment fields such as lead time become available. This would be significant for the adoption of any no-show or wait-time model that seeks to prevent unfair biases in prediction by producing a demographically blind model (Taheri-Shirazi et al., 2023).

Finally, the thresholding results prove to be the most promising display of use for our predictive models. A clinical deployment of these classifications into a stochastic optimization algorithm would likely utilize the predicted probabilities as contingencies for different protocols. If a patient is predicted to have a very high risk of no-show or long wait-time, more invasive protocols may be suggested. This makes model calibration an important aspect when selecting a final model beyond just the accuracy and precision metrics. The calibration results revealed that the LLM and XGBoost remained well calibrated on unseen data with the LLM being more robust in its predictions. Furthermore, the results show that XGBoost strikes the best balance for the number of high probability predictions and its high precision for no-shows while the BERT model provided the best precision using a 90% threshold for waiting times.

### 4.1 Limitations

Our study was limited in several ways while leaving room for future work. Firstly, we were unable to utilize lead-time data for no-show appointments due to the nature of data collection in the scheduling system. This may have prevented even larger improvements in performance based on its significance in literature. Next, the clinician specialties were estimated substitutes for a field called “ordering department” since the current system overwrites the original clinical department with the modality upon completion of the exam. With regards to our ClinicalBERT LLM models, although we demonstrated the viability of this architecture for these tasks, significant improvements can still be made. These may include more contextual input data, fine tuning the rebalancing techniques with thresholding, more sophisticated input data augmentation, and experimenting with other large foundational models such as GPT models. Furthermore, the reason for exam feature is dependent on providers incorporating meaningful comments to contextualize the appointment. Additionally, our models did not directly account for missingness, which is a common issue in medical records data. The models assume features required in training are present in test data. Future work should attempt to generalize the models further by testing on external data while verifying performance on data with missing values. This would improve robustness and applicability for other institutions. Finally, the target no-show event had a significantly smaller set of data that included a proper reason for exam. In the future, if an extensive audit can be carried out, more contextual information regarding the reason for the appointment could prove useful when predicting no-shows.

## 5 Conclusion

Our research tackled two important use cases which are to survey the best models and methods to predict patient no-show and long wait-times. The study not only improved prediction metrics given a significantly smaller event rate compared to other studies, but it also revealed a new area of potential advancements in classification on these tasks with LLMs, allowing for effective use of more unstructured data to further improve predictions. Our research has also shown that contextual appointment related data such as a “reason for exam” and scan priority or modality, are more useful in predicting appointment outcome than patient demographics. This would promote greater equity when implementing a proposed AI scheduler by producing a demographically blind model that predicts based on appointment features rather than the patient’s postal demographics. Finally, using a series of varied models and data balancing techniques, we found that for no-shows, the XGBoost performed the best when accounting for both AUC and F1 while the RF and BERT models would be the best choices for a wait-time model. Our future work will utilize continuous probability predictions for a downstream AI assisted scheduling system to reduce these event related costs and provide targeted accommodations for individuals more likely to experience these events.

## Data Availability

The datasets presented in this article are not readily available due to the hospital’s policy. Requests to access the datasets should be directed to farzad.khalvati@utoronto.ca. Code for algorithms and analysis can be found using the following github repository, https://github.com/IMICSLab/Predicting_DIR_no_shows_and_long_wait_times. This will be updated with applicable code but will not include any trained weights or data.

## Appendix. Data processing and feature engineering

This appendix provides additional information on the exact feature sets used within our model training before and after cleaning. It also provides a sample prompt used for our LLM training input.

### Raw feature list after initial cleaning

The following is a list of our final feature after cleaning: ‘AnonymizedID,’ ‘ORDER_ID,’ ‘Modality,’ ‘PATIENT_CLASS,’ ‘ORDERING_DTTM,’ ‘ORDERING_DEPARTMENT_NAME,’ ‘SCHED_ON_DTTM,’ ‘SCHED_EXAM_DTTM,’ ‘CHECKIN_DTTM,’ ‘age_at_scan,’ ‘procedure_name,’ ‘APPT_NAME,’ ‘week_day,’ ‘scheduled_hour,’ ‘no_show,’ ‘distance,’ ‘pre_appointments,’ ‘pre_no_show,’ ‘income,’ ‘non_eng_%,’ ‘lone_parent_%,’ ‘GA,’ ‘CARE_COORDITION_FLAG_YN,’ ‘WTIS_PRIORITY,’ ‘WTIS_SDP,’ ‘appointment_duration,’ ‘wait_time,’ ‘gender_numeric,’ ‘DistanceInKMFromSK,’ ‘REASON_FOR_EXAM,’ ‘postal,’ ‘Modality.1,’ ‘Order_Creator,’ ‘ORDERING_PROVIDER,’ ‘AUTHORIZING_PROVIDER,’ ‘TECHNOLOGIST_NAME,’ ‘REASN_FOR_EXAM,’ ‘Authorizing Category,’ ‘Re-Clean Flag,’ ‘lead_time,’ ‘lead_time_bins,’ ‘exam_time_of_day,’ ‘sched_month,’ ‘sched_year,’ ‘sched_month_number,’ ‘postal_encoded,’ ‘Specs_encoded’.

### Final feature set used for training

The following list are the features used in training: ‘age_at_scan,’ ‘procedure_name,’ ‘week_day,’ ‘scheduled_hour,’ ‘distance,’ ‘pre_appointments,’ ‘pre_no_show,’ ‘income,’ ‘non_eng_%,’ ‘lone_parent_%,’ ‘GA,’ ‘CARE_COORDITION_FLAG_YN,’ ‘WTIS_PRIORITY,’ ‘WTIS_SDP,’ ‘gender_numeric,’ ‘sched_year,’ ‘sched_month_number,’ ‘postal_encoded,’ ‘Specs_encoded,’ ‘appointments_in_hour.’

### Sample prompt for LLM model input

The following sentence is a fake example (for data privacy reasons) of the kind of input we used in this model with each input following the same structure with different values included from the columns:

“A male pediatric patient requires a diagnostic appointment for a MRI scan which was referred by a clinician specializing in Neurosurgery. The patient will be 2000 days old at the time of the appointment, which is currently scheduled for Thursday June 1, 2018 at 4 h. This appointment is of top priority. The patient’s parental household income is estimated as $31280.0 CAD. The patient comes from a neighborhood with a 21.862% single parent demographic. This neighborhood also has a 6.995% non-English speaking demographic. The address postal code is L1A with a distance of 50.26 KM from the Hospital. The patient also has a history of not showing up to 0 appointments out of the 1 previous appointment they have had.”
